# State-Level Promotion of Plant-Based School Meals in the United States: A National Policy Review †

**DOI:** 10.3390/nu18152556

**Published:** 2026-08-05

**Authors:** Vishakha Modak, Aditya Karthikeyan, Natali Sorajja, Sridhar Mangalesh, Priyansh Shah, Sharanya Kaushik, Eleonora Gashi, Robert Faillace

**Affiliations:** Jacobi Medical Center, Albert Einstein College of Medicine, New York City Health + Hospitals, 1400 Pelham Parkway, Bronx, NY 10461, USA; adk300399@gmail.com (A.K.); natali.sorajja@einsteinmed.edu (N.S.); mailsmangalesh@gmail.com (S.M.); drpriyanshshah@gmail.com (P.S.); sharanya.kaushik3@gmail.com (S.K.); gashibe@nychhc.org (E.G.); robert.faillace@nychhc.org (R.F.)

**Keywords:** plant-based diet, school lunch, pediatric nutrition, cardiometabolic health, public health policy, vegetarian, USDA, National School Lunch Program

## Abstract

**Background:** Pediatric cardiometabolic health in the United States is declining, driven by rising rates of obesity, prediabetes, and type 2 diabetes. School lunch programs provide a unique opportunity to influence children’s dietary behaviors, reaching millions of students across diverse socioeconomic backgrounds each day. Increasing the availability of plant-based meals in school lunch programs may help reduce modifiable cardiometabolic risk factors and serve as a cost-effective, population-level strategy to promote long-term cardiovascular health. **Methods:** We conducted a cross-sectional narrative policy review of school lunch programs across all 50 U.S. states and the District of Columbia. Publicly available state statutes, regulations, school nutrition guidance, government reports, and educational resources were systematically reviewed to identify official statewide policies or guidance explicitly supporting plant-based or vegetarian meals. Jurisdictions were classified as providing either Explicit Support or Not Specified according to predefined coding criteria. We also assessed whether jurisdictions specified the frequency of plant-based meal offerings. **Results:** Only 3 of 51 jurisdictions (5.9%)—California, the District of Columbia, and Illinois—provided explicit statewide support for plant-based meal options. The remaining 48 jurisdictions (94.1%) did not explicitly promote or support plant-based meals in official statewide school nutrition policies or guidance. No jurisdiction mandated the frequency of plant-based meal offerings, although the District of Columbia provided non-binding guidance encouraging regular inclusion. **Conclusions:** Explicit statewide support for plant-based or vegetarian school meals remains rare in the United States. Strengthening state-level policies and developing standardized guidance on the inclusion and frequency of plant-based meals in school lunch programs may represent a feasible population-level strategy to improve dietary quality and reduce future pediatric cardiometabolic risk.

## 1. Introduction

Poor diet quality remains a leading driver of cardiovascular disease. The 2026 American Heart Association (AHA) dietary guidance emphasizes heart-healthy dietary patterns beginning early in life and sustained across the life course, including adequate intake of vegetables, fruits, and whole grains and a shift from meat to plant proteins such as legumes and nuts [1]. These recommendations encompass both plant-forward dietary patterns, which emphasize plant foods without necessarily excluding animal products, and plant-based meals, which emphasize foods derived from plants—including vegetables, fruits, whole grains, legumes, nuts, and seeds—and may include vegetarian or vegan meal options. Recent evidence suggests that healthy plant-based diets in children and adolescents are associated with improved diet quality and favorable cardiometabolic outcomes, including lower LDL cholesterol, healthier body composition, and reduced prevalence of metabolic syndrome. These findings support the implementation of healthy plant-based dietary patterns via school meals.

Federal dietary guidance similarly supports nutrient-dense dietary patterns with a variety of protein sources, including plant-sourced foods. The 2024 U.S. Department of Agriculture (USDA) school meal rule was based on the 2020–2025 Dietary Guidelines for Americans, and the 2025–2030 update continues to emphasize whole, nutrient-dense foods and plant-sourced proteins [2,3].

School meals offer a high-reach setting for shaping children’s diets. In the 2023–2024 school year, nearly 29.4 million children participated in the National School Lunch Program (NSLP), including 21.1 million who received free or reduced-price lunch [4]. Participation in the NSLP has been associated with greater consumption of fruits, vegetables, and whole grains and better overall diet quality compared with non-participation [5].

The 2024 USDA rule introduced menu-planning flexibilities expanding options for nuts, seeds, and other plant-based protein sources, thereby increasing options for schools offering vegetarian and other plant-based meal options. However, schools are not required to change operations or offer such meals, and federal permissibility leaves substantial discretion to states and districts [6]. Beyond their potential cardiometabolic benefits, plant-based school meals may also contribute to broader public health goals, including environmental sustainability, dietary equity through more inclusive meal options, and food system resilience.

We conducted a national policy review of all 50 states and the District of Columbia to characterize jurisdiction-level support for plant-forward school lunch options and whether policies specify their frequency.

## 2. Methods

We conducted a cross-sectional narrative policy review of publicly available school meal policies and guidance across all 50 U.S. states and the District of Columbia. Searches were conducted from September 2025 through 31 January 2026, using official state education, child nutrition, and government websites. To maximize retrieval, searches were performed both directly within official government websites and using external search engines. Search terms included combinations of “plant-based,” “vegetarian,” “vegan,” “meatless,” “school lunch,” “school meals,” “child nutrition,” and “menu planning.” Relevant linked policy documents, legislative text, implementation guidance, and program materials were also reviewed. Source hyperlinks for all jurisdictions are provided in Supplementary File S1.

For each jurisdiction, the source, policy or program name, description of plant-based or vegetarian meal support, implementation status (enacted or proposed), and any stated frequency of meal offerings were extracted.

Documents identified through this standardized search process were independently classified by two reviewers (A.K. and V.M.) into one of two mutually exclusive categories (Table 1):

- Explicit Support: Enacted statewide policies or official statewide guidance explicitly promoting or supporting plant-based meals.
- Not Specified: No enacted statewide policy or official statewide guidance explicitly promoting or supporting plant-based school meals. This category included jurisdictions with only USDA compliance, Farm-to-School or local procurement initiatives, proposed or unenacted legislation, pilot programs, district-level initiatives, or other non-statewide efforts.

When multiple documents were available for jurisdiction, classification was based on the highest level of enacted statewide policy or official statewide guidance. If information was ambiguous or conflicting, primary legislative or state agency documents were prioritized over secondary program materials.

Discrepancies between reviewers were resolved by consensus after re-review of the primary source documents. Inter-rater agreement was assessed using Cohen’s κ prior to consensus discussion; the overall κ was 0.82, indicating strong agreement.

This policy review relied exclusively on publicly available governmental documents. No human subjects were involved, and Institutional Review Board approval was not required.

## 3. Results

**Figure 1:**
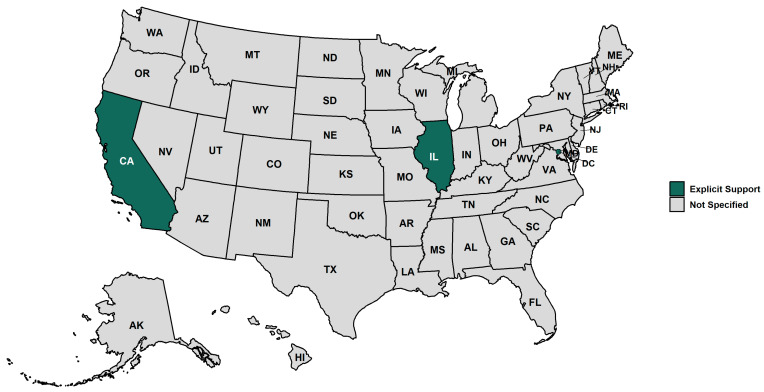
Choropleth map showing jurisdiction-level classification of statewide plant-based school meal policy support across the 50 U.S. states and the District of Columbia. Colors indicate classification: *green* = Explicit Support (*n* = 3: California, District of Columbia, Illinois); *gray* = Not Specified (*n* = 48). State abbreviations: AL: Alabama; AK: Alaska; AZ: Arizona; AR: Arkansas; CA: California; CO: Colorado; CT: Connecticut; DC: District of Columbia; DE: Delaware; FL: Florida; GA: Georgia; HI: Hawaii; ID: Idaho; IL: Illinois; IN: Indiana; IA: Iowa; KS: Kansas; KY: Kentucky; LA: Louisiana; ME: Maine; MD: Maryland; MA: Massachusetts; MI: Michigan; MN: Minnesota; MS: Mississippi; MO: Missouri; MT: Montana; NE: Nebraska; NV: Nevada; NH: New Hampshire; NJ: New Jersey; NM: New Mexico; NY: New York; NC: North Carolina; ND: North Dakota; OH: Ohio; OK: Oklahoma; OR: Oregon; PA: Pennsylvania; RI: Rhode Island; SC: South Carolina; SD: South Dakota; TN: Tennessee; TX: Texas; UT: Utah; VT: Vermont; VA: Virginia; WA: Washington; WV: West Virginia; WI: Wisconsin; WY: Wyoming.

Among the 51 jurisdictions evaluated, 3 (5.9%) were classified as providing Explicit Support for plant-based school meals: California, the District of Columbia, and Illinois (Figure 1). California provides official statewide guidance supporting plant-based meal options. The District of Columbia enacted legislation requiring schools to provide a vegetarian main-course option daily and encouraging schools to serve plant-based food options. Illinois enacted statewide legislation requiring schools participating in the National School Lunch Program to make a plant-based lunch option available upon request. Additional policy details and source documents are provided in Supplementary File S1.

The remaining 48 jurisdictions (94.1%) were classified as Not Specified, indicating that no enacted statewide policy or official statewide guidance explicitly promoting or supporting plant-based or vegetarian school meals was identified. This category was heterogeneous and included jurisdictions that adhered to USDA meal-pattern requirements without additional statewide guidance, promoted Farm-to-School or local procurement initiatives, had proposed or unenacted legislation, implemented pilot or grant-funded initiatives, or described district-level efforts that were not adopted statewide (Supplementary File S1). For example, although New York City has implemented initiatives such as Meatless Mondays and Plant-Powered Fridays, these are district-level programs rather than statewide policies and therefore did not meet the criteria for Explicit Support.

Regarding meal frequency, no jurisdiction mandated a minimum frequency for plant-based meal offerings. Although the District of Columbia requires schools to provide a vegetarian food option as the main course for breakfast and lunch every day and encourages schools to serve plant-based food options as the main course at breakfast and lunch each day, California and Illinois do not specify the frequency with which plant-based meal options must be offered.

## 4. Discussion

In this review, only 3 of 51 U.S. jurisdictions had enacted statewide policies or official guidance explicitly supporting plant-based meals. The remaining 48 jurisdictions were classified as “Not Specified,” indicating that no qualifying statewide policy was identified through publicly available governmental sources. Several jurisdictions, including Hawaii, Minnesota, and New Jersey, have proposed legislation or initiatives supporting plant-based school meals; however, these have not been enacted at the statewide level. Although no jurisdiction specified a minimum frequency for plant-based meal offerings, this finding indicates that existing statewide policies generally address meal availability rather than how often plant-based options should be offered.

These findings suggest that federal permissibility for plant-based meals has not translated into consistent statewide policy support. This variation is notable in the context of the AHA 2026 Scientific Statement and the 2025–2030 Dietary Guidelines for Americans, both of which encourage greater consumption of plant-derived foods as part of healthy dietary patterns [1,3]. These findings should also be considered within the broader international context. The EAT–Lancet Commission (2025) identifies plant-rich dietary patterns as a key strategy for promoting both human and planetary health and recognizes schools as an important setting for supporting healthier food systems [7]. Consistent with this approach, France requires at least one vegetarian meal per week in school canteens, while Belgium has demonstrated the feasibility of plant-forward school meals through initiatives such as Ghent’s “Thursday Veggie Day.” [8,9]. Similar district-level initiatives have emerged in the United States, including New York City’s Meatless Mondays and Plant-Powered Fridays; however, these have not been adopted through statewide policy. A recent analysis of National School Lunch Program meals found that school lunches were not fully aligned with the EAT–Lancet healthy reference diet, with lower-than-recommended amounts of whole grains, legumes, nuts, and vegetables and higher amounts of refined grains, dairy, and red meat [10]. Together, these findings highlight opportunities to strengthen school meal policies. They also provide a foundation for future research evaluating policy implementation, accessibility, meal frequency, school food environments, and children’s dietary intake.

This study has limitations. It relies on publicly available governmental webpages and documents and may not have captured all relevant policy documents, administrative guidance, procurement resources, or district-level initiatives. Additionally, jurisdictions classified as “Not Specified” may have additional administrative guidance or local implementation practices that were not reflected in publicly available statewide governmental documents. This review evaluates policy availability rather than implementation and did not assess school menus, student meal selection, dietary intake, or health outcomes. Consequently, the presence of a policy does not necessarily indicate that students consume more plant-based meals. Finally, the search concluded on 31 January 2026, and subsequent policy developments were not captured.

Despite these limitations, this review demonstrates substantial variation in publicly available statewide policy support for plant-based school meals across the United States. By providing a transparent overview of the current policy landscape, these findings establish a foundation for future studies examining policy implementation and whether stronger statewide support is associated with healthier school food environments and improved dietary patterns among children.

## Figures and Tables

**Table 1:** Coding framework used to classify U.S. jurisdiction-level policies related to plant-based school lunch programs.

Domain	Label	Definition
**Level of Official Support**	Explicit Support	Enacted statewide policy or official statewide guidance explicitly promoting or supporting plant-based meals.
	Not Specified	No enacted statewide policy or official statewide guidance explicitly promoting or supporting plant-based school meals; includes USDA-compliance-only, Farm-to-School/local procurement, proposed or unenacted legislation, pilot programs, or district-level efforts not adopted statewide.
**Frequency of Offerings**	Days/Week (mandated frequency)	The jurisdiction mandates/requires schools to serve plant-based meals a specific number of times per week. If no mandate exists, coded as “None mandated.”

Note: Jurisdictions were classified into two mutually exclusive categories, Explicit Support (*n* = 3) and Not Specified (*n* = 48). Hawaii, Minnesota, and New Jersey are counted within the Not Specified category, as their proposed policies had not been enacted statewide by the search close date (31 January 2026).

## Data Availability

All policy documents reviewed are publicly available via official state and federal government websites. Source hyperlinks are provided in Supplementary File S1 (eMethods).

## Supplementary Materials

The following supporting information can be downloaded at https://www.mdpi.com/article/10.3390/nu18152556/s1, Supplementary File S1 (eMethods)—hyperlinked policy sources for all 51 jurisdictions reviewed, including the URL, access date, policy/program name, and evidence basis for each jurisdiction’s classification.
