# Supplementary material for: State-Level Promotion of Plant-Based School Meals in the United States: A National Policy Review
Source: Nutrients. 2026 Aug 5;18(15):2556. doi: 10.3390/nu18152556 (PMC13468650; doi:10.3390/nu18152556)
Supplement: Supplementary file 1 [file nutrients-18-02556-s001.zip › Supplementary File S1 (eMethods).pdf]

Online Supplementary Material

State Level Promotion of Plant-Based School Meals in the United States: A National Policy Review

eMethods: Jurisdiction-level classification, basis for classification, and source hyperlinks

Classification follows the two-category scheme defined in Table 1 of the manuscript: Explicit Support (enacted statewide legislation, regulation, or official statewide policy/guidance explicitly promoting, encouraging, or requiring plant-based or vegetarian school meal options) versus Not Specified (no such enacted statewide measure identified, including jurisdictions with Farm-to-School programs, local food procurement initiatives, universal meal programs, proposed-but-unenacted legislation, professional-development/training resources, or district-/city-level initiatives without statewide implementation). The Basis for Classification column records the specific type of evidence underlying each jurisdiction's classification, to make the heterogeneity within the Not Specified category transparent.

Serial Number	State Name	Classification	Basis for Classification (Evidence Type)	Hyperlink(s)
1	Alabama	Not Specified	Statewide produce-promotion / Farm-to-School initiatives only; no plant-based or vegetarian meal policy identified.	https://www.alabamapublichealth.gov/npa/more-matters.html<br>https://www.alabamapublichealth.gov/npa/index.html
2	Alaska	Not Specified	No relevant statewide policy identified.	https://education.alaska.gov/SchoolFinance/ChildNutrition
3	Arizona	Not Specified	Statewide materials follow USDA meal standards only; no plant-based/vegetarian promotion.	https://www.azed.gov/nutrition
4	Arkansas	Not Specified	Farm-to-School / local food initiatives only; no plant-based/vegetarian promotion.	https://dese.ade.arkansas.gov/Offices/educator-effectiveness/child-nutrition
5	California	**Explicit Support**	Statewide guidance and resolution (ACR 16) plus dedicated funding encouraging daily plant-based meal availability.	https://www.cde.ca.gov/ls/nu/he/vegmealoptionscnp.asp
6	Colorado	Not Specified	Local food purchasing incentives (Healthy School Meals for All) only; no plant-based/vegetarian promotion.	https://cde.state.co.us/nutrition/osnnslpmealpattern
7	Connecticut	Not Specified	Vegetarian resources limited to dietary accommodation, not routine menu policy.	https://portal.ct.gov/SDE/Nutrition/Program-Guidance/Program-Guidance-for-School-Nutrition-Programs
8	Delaware	Not Specified	Farm-to-School / local food procurement only; no plant-based/vegetarian promotion.	https://education.delaware.gov/child-nutrition/

Serial Number	State Name	Classification	Basis for Classification (Evidence Type)	Hyperlink(s)
9	District of Columbia	**Explicit Support**	Requires a daily vegetarian main-course option at breakfast and lunch, with daily rotation/labeling.	https://osse.dc.gov/page/school-meals
10	Florida	Not Specified	Farm to School Program and vegetarian menu-planning resources; no explicit statewide promotion.	http://www.floridahealth.gov/programs-and-services/community-health/school-health/index.html
11	Georgia	Not Specified	Farm-to-School / menu-planning resources include vegetarian considerations; no explicit statewide promotion.	https://www.decal.ga.gov/
12	Hawaii	Not Specified	Proposed legislation / pilot initiatives; not enacted as statewide policy.	https://data.capitol.hawaii.gov/sessions/session2025/bills/HB328_HD1_.pdf
13	Idaho	Not Specified	Farm to School Program (local procurement, nutrition education) only.	https://sde.idaho.gov/about-us/departments/child-nutrition-programs/school-meal-programs/fresh-fruit-vegetable-program/
14	Illinois	**Explicit Support**	Statewide law requiring districts to provide a plant-based lunch option to students who submit a prior request.	https://dph.illinois.gov/topics-services/community-health/school-health.html
15	Indiana	Not Specified	Farm to School Program only; no plant-based/vegetarian promotion.	https://www.in.gov/doe/nutrition/foods-distribution-program/
16	Iowa	Not Specified	No relevant statewide policy identified.	https://educate.iowa.gov/pk-12/operation-support/nutrition-programs/ffvp
17	Kansas	Not Specified	Farm to Plate / Farm to School initiatives only.	https://cnw.ksde.gov/
18	Kentucky	Not Specified	Farm to School Program only.	https://education.ky.gov/federal/SCN/Pages/School-Meal-Programs.aspx
19	Louisiana	Not Specified	Source covers federal nutrition programs (NSLP/SBP/CACFP/SFSP), Farm to School, and local nutrition-education initiatives only; no plant-based/vegetarian promotion identified.	https://www.louisianabelieves.com/resources/library/child-nutrition-programs
20	Maine	Not Specified	Farm and Sea to School, Local Foods Fund and related local-food-procurement programs only.	https://www.maine.gov/doe/nutrition

Serial Number	State Name	Classification	Basis for Classification (Evidence Type)	Hyperlink(s)
21	Maryland	Not Specified	Farm-to-School Meal Grant Program (local procurement) only.	http://marylandpublicschools.org/about/Pages/DCAA/Child-Nutrition.aspx
22	Massachusetts	Not Specified	Nutrition Standards Commission recommended daily vegan/vegetarian options as a long-term goal, but this is a recommendation, not enacted policy.	http://www.doe.mass.edu/cnp/
23	Michigan	Not Specified	10 Cents a Meal / Farm to School (local procurement); plant-based resources available but no explicit statewide promotion.	https://www.michigan.gov/mde/0,4615,7-140-6583_6503_59124---,00.html
24	Minnesota	Not Specified	Proposed legislation (HF 2764) would require plant-based meal options; not enacted.	https://caa.mn/2025/08/18/minnesota-takes-an-important-step-forward-on-plant-based-school-meals/
25	Mississippi	Not Specified	Farm to School Program only.	https://www.mdek12.org/sites/default/files/documents/MBE/State%20Board%20Policy/Chapter%2038/rule_38.11_final_1.5.2023.pdf
26	Missouri	Not Specified	Farm-to-School / Farm-to-Table (local procurement) initiatives only.	https://dese.mo.gov/financial-admin-services/food-nutrition-services/modifications-use-vegetable-protein-product
27	Montana	Not Specified	Farm to School Program (local procurement, agricultural education) only.	https://www.montana.edu/teamnutrition/wellness/
28	Nebraska	Not Specified	Farm to School initiatives (local procurement) only.	https://www.education.ne.gov/ns/nslp/fresh-fruit-and-vegetable-program/
29	Nevada	Not Specified	Farm to School Program only.	https://nutrition.nv.gov/Programs/Wellness_documents/Wellness_Policies/NV_Wellness_Policy_2014_07_14/
30	New Hampshire	Not Specified	Farm to School Program (local procurement, nutrition education) only.	https://www.education.nh.gov/who-we-are/division-of-learner-support/bureau-wellness-and-nutrition/office-nutrition-programs-and-services-onps/fresh-fruit-and-vegetable-program
31	New Jersey	Not Specified	Proposed legislation (S1676) would require at least one daily plant-based option; not enacted.	https://www.nj.gov/agriculture/divisions/fn/childadult/school_lunch.shtml
32	New Mexico	Not Specified	Universal school meal access (Healthy Hunger-Free Students' Bill); no explicit plant-based/vegetarian promotion.	https://web.ped.nm.gov/wp-content/uploads/2025/06/2025-Healthy-Universal-School-Meals-Program-Guidance-FINAL.pdf
33	New York	Not Specified	No statewide policy identified; NYC's “Plant-Powered Friday” is a municipal/district program, not a statewide policy.	https://www.schools.nyc.gov/school-life/food/school-meals/plant-powered
34	North Carolina	Not Specified	Source covers federal meal-pattern rules and Offer-vs-Serve	https://www.dpi.nc.gov/districts-schools/district-operations/school-nutrition/information-resources-subject/meal-planning-production-school-meals

Serial Number	State Name	Classification	Basis for Classification (Evidence Type)	Hyperlink(s)
			guidance; a one-time plant-based recipe training webinar was offered to nutrition staff, but no statewide policy or regulation promoting plant-based/vegetarian meals was identified.	
35	North Dakota	Not Specified	Farm to School Program only.	https://www.nd.gov/dpi/districtsschools/child-nutrition-and-food-distribution
36	Ohio	Not Specified	Farm to School Program only.	https://education.ohio.gov/Topics/Student-Supports/Food-and-Nutrition
37	Oklahoma	Not Specified	Farm to School Program only.	https://oklahoma.gov/education/services/child-nutrition.html<br>https://oklahoma.gov/content/dam/ok/en/osde/documents/services/child-nutrition/child-nutrition-documents/school-meal-program-various-documents-forms/2026%20School%20Training%20Manual.pdf
38	Oregon	Not Specified	Student Success Act / Farm to Child Nutrition Programs (universal access, local procurement) only.	https://www.ode.state.or.us/wma/nutrition/snp/pages/default.aspx<br>https://www.oregon.gov/ode/students-and-family/childnutrition/snp/pages/meal-pattern-and-nutritional-quality.aspx
39	Pennsylvania	Not Specified	Farm to School Grant Program / Network (local procurement) only.	https://www.pa.gov/agencies/education/programs-and-services/schools/food-and-nutrition/programs/national-school-lunch-program
40	Rhode Island	Not Specified	Farm to School initiatives (local procurement) only.	https://ride.ri.gov/child-nutrition/nutrition-programs/national-school-lunch-program
41	South Carolina	Not Specified	Farm to School Program only.	https://ed.sc.gov/districts-schools/health-and-nutrition/meal-programs/national-school-lunch-program/
42	South Dakota	Not Specified	Farm to School Program only.	https://doe.sd.gov/cans/
43	Tennessee	Not Specified	Farm to School Program only.	https://www.tn.gov/education/districts/snp-resources/snp-programs.html
44	Texas	Not Specified	Farm Fresh Initiative / Farm Fresh Challenge (local procurement) only.	https://www.dshs.texas.gov/schoolhealth
45	Utah	Not Specified	Farm to Fork Program (local procurement) only.	https://schools.utah.gov/cnp
46	Vermont	Not Specified	Farm to School & Early Childhood Program (local procurement, school gardens) only.	https://education.vermont.gov/student-learning/nutrition-programs
47	Virginia	Not Specified	Farm to School Program only.	http://www.doe.virginia.gov/support/nutrition/index.shtml
48	Washington	Not Specified	Farm to School Program only.	https://www.k12.wa.us/student-success/health-safety/child-nutrition
49	West Virginia	Not Specified	Farm to School Program only.	https://wvde.us/child-nutrition-services/
50	Wisconsin	Not Specified	Farm to School Program only.	https://dpi.wi.gov/school-nutrition
51	Wyoming	Not Specified	Farm to School Program only.	https://edu.wyoming.gov/services/child-nutrition/
